# Actomyosin-Assisted Pulling of Lipid Nanotubes from Lipid Vesicles and Cells

**DOI:** 10.1021/acs.nanolett.1c04254

**Published:** 2022-01-28

**Authors:** Kevin Jahnke, Stefan J. Maurer, Cornelia Weber, Jochen Estebano Hernandez Bücher, Andreas Schoenit, Elisa D’Este, Elisabetta Ada Cavalcanti-Adam, Kerstin Göpfrich

**Affiliations:** † Biophysical Engineering Group, Max Planck Institute for Medical Research, Jahnstraße 29, D-69120 Heidelberg, Germany; ‡ Department of Physics and Astronomy, Heidelberg University, D-69120 Heidelberg, Germany; § Department of Cellular Biophysics, Max Planck Institute for Medical Research, Jahnstraße 29, D-69120 Heidelberg, Germany; ∥ Optical Microscopy Facility, Max Planck Institute for Medical Research, Jahnstraße 29, D-69120 Heidelberg, Germany

**Keywords:** Lipid nanotubes, lipid tether pulling, motility assay, giant unilamellar vesicle, membrane-to-cortex attachment, actin, heavy mero-myosin

## Abstract

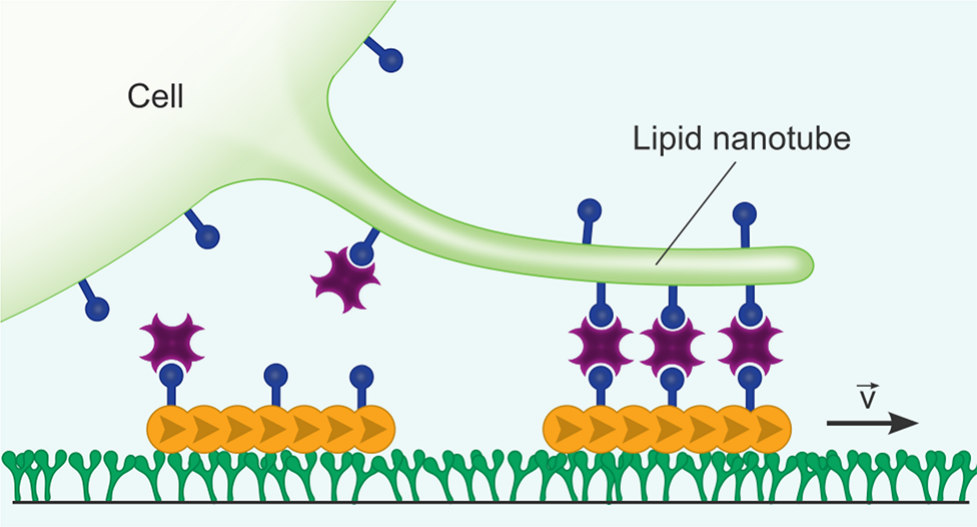

Molecular motors are pivotal for intracellular transport as well as cell motility and have great potential to be put to use outside cells. Here, we exploit engineered motor proteins in combination with self-assembly of actin filaments to actively pull lipid nanotubes from giant unilamellar vesicles (GUVs). In particular, actin filaments are bound to the outer GUV membrane and the GUVs are seeded on a heavy meromyosin-coated substrate. Upon addition of ATP, hollow lipid nanotubes with a length of tens of micrometer are pulled from single GUVs due to the motor activity. We employ the same mechanism to pull lipid nanotubes from different types of cells. We find that the length and number of nanotubes critically depends on the cell type, whereby suspension cells form bigger networks than adherent cells. This suggests that molecular machines can be used to exert forces on living cells to probe membrane-to-cortex attachment.

Molecular motors govern various cellular processes from intracellular transport to contraction and cell division. While the development of artificial macroscale motors flourishes, many biophysical goals benefit from the engineering of motors on the nanoscale.^1,2^ In particular, man-made machines like optical tweezers or atomic force microscopes have long been employed to probe cellular properties like membrane-to-cortex adhesion,^3^ yet the use of molecular machines for this purpose remains largely unexplored. To date, natural motor proteins have been used to deform giant vesicles,^4−7^ assemble contractile systems,^8−10^ or transport cargo.^11−15^ Moreover, noteworthy efforts have been made to build synthetic nanoscale motors with DNA nanotechnology as transporters,^16,17^ rotors^18^ or sliders.^19^ However, due to their comparably low processivity and force generation compared to natural motors, the amount of suitable applications for synthetic motors is still limited. On the other hand, recent progress has been made using a minimal system of vesicles and natural motor proteins to elucidate the complex interplay of membrane tubulation of synthetic vesicles^20,21^ and membrane dynamics.^22^ Still, it remains elusive and uncertain how these minimal systems perform in more complex environments of natural cells and if they can possibly provide direct evidence of a cell’s biophysical properties or even guide cell functions.

Here, we develop a minimal system to actively pull lipid nanotubes from giant unilamellar vesicles (GUVs) and natural cells. Lipid nanotubes are membrane-enclosed tubes with nanoscale diameters that can guide the transfer of vesicles and organelles between cells.^23^ We analyze the network length per cell and find that it critically depends on the cell type. This indicates that our minimal motor-based system could be used as a straightforward method to probe membrane-to-cortex attachment as a crucial biophysical indicator for the cell state.^3^

First, we set out to establish a motor-based force-generating system that can be used to actively pull lipid nanotubes. This requires a mechanism for directional force generation. For this purpose, we engineer two variants of an *in vitro* actin motility assay as illustrated in Figure 1a. First, the substrate is functionalized with a truncated version of myosin consisting of only the functional headgroup of myosin II. This so-called heavy meromyosin (HMM) is capable of performing a power stroke like myosin II but is still easily soluble in water at physiological conditions. We purify the HMM and actin from rabbit skeletal muscle and verify the successful purification with denaturing polyacrylamide gel electrophoresis (SDS-PAGE, Supporting Information (SI) Figure S1). The HMM is immobilized on the substrate where it binds prepolymerized actin filaments to the substrate and translocates them in the presence of adenosine triphosphate (ATP) like in a conventional motility assay.^24^ At low actin concentrations from 0.5 to 20 nM, we observe the attachment and random movement of individual actin filaments, termed random filaments, with confocal microscopy (Figure 1b, SI Video S1). In order to make the movement of actin filaments directional, we induce the nematic ordering of actin filaments by adding a high concentration (24 μM) of unlabeled F-actin to the motility assay. We thereby surpass the critical filament density ρ_*c*_ of 5 filaments/μm^2^ above which filament ordering takes place.^25^ This causes the alignment and movement of actin filaments on parallel tracks (Figure 1b, SI Video S2). With particle image velocimetry (PIV), we obtain the velocity vector field which yields a correlation length of 6.7 ± 3.9 μm for random filaments and 29.1 ± 12.5 μm for aligned filaments (Figure 1c).^26^ From the confocal images (Figure 1b) and the corresponding velocity vector field (Figure 1c), we verify the alignment of actin filaments within equidistant filament streams with a spacing of 3.6 ± 1.4 μm. We analyze the orientation, that is, the direction of the velocity vector, of individual actin filaments over time and find that in the standard *in vitro* actin motility assay the filaments move in random directions (Figure 1d), whereas they move along one axis with a strong bias to one direction when the actin was nematically aligned. For the aligned condition, some filaments move in the 180° opposing direction and swirls and vortices can occur inducing a global change in the direction of the bias (SI Figure S2 and Video S3).^27^ To probe the effect that the alignment has on the actin filament velocity, we quantified the average filament velocity for both, random and aligned actin at room temperature (RT) and 30 °C (Figure 1e), which is closer to the optimum temperature for HMM activity.^28^ The velocity for random filaments at RT is significantly smaller than at 30 °C. Additionally, the alignment significantly enhances the average velocity of actin filaments from 0.9 ± 0.9 to 2.0 ± 1.0 μm s^–1^ (*p* ≤ 0.0001). This might be due to the existence of defined tracks for aligned actin that allow for a higher motor processivity compared to when actin filaments are randomly distributed. Additionally, dysfunctional HMM may be blocked by unlabeled actin filaments increasing the overall actin filament velocity.^29^

**Figure 1:**
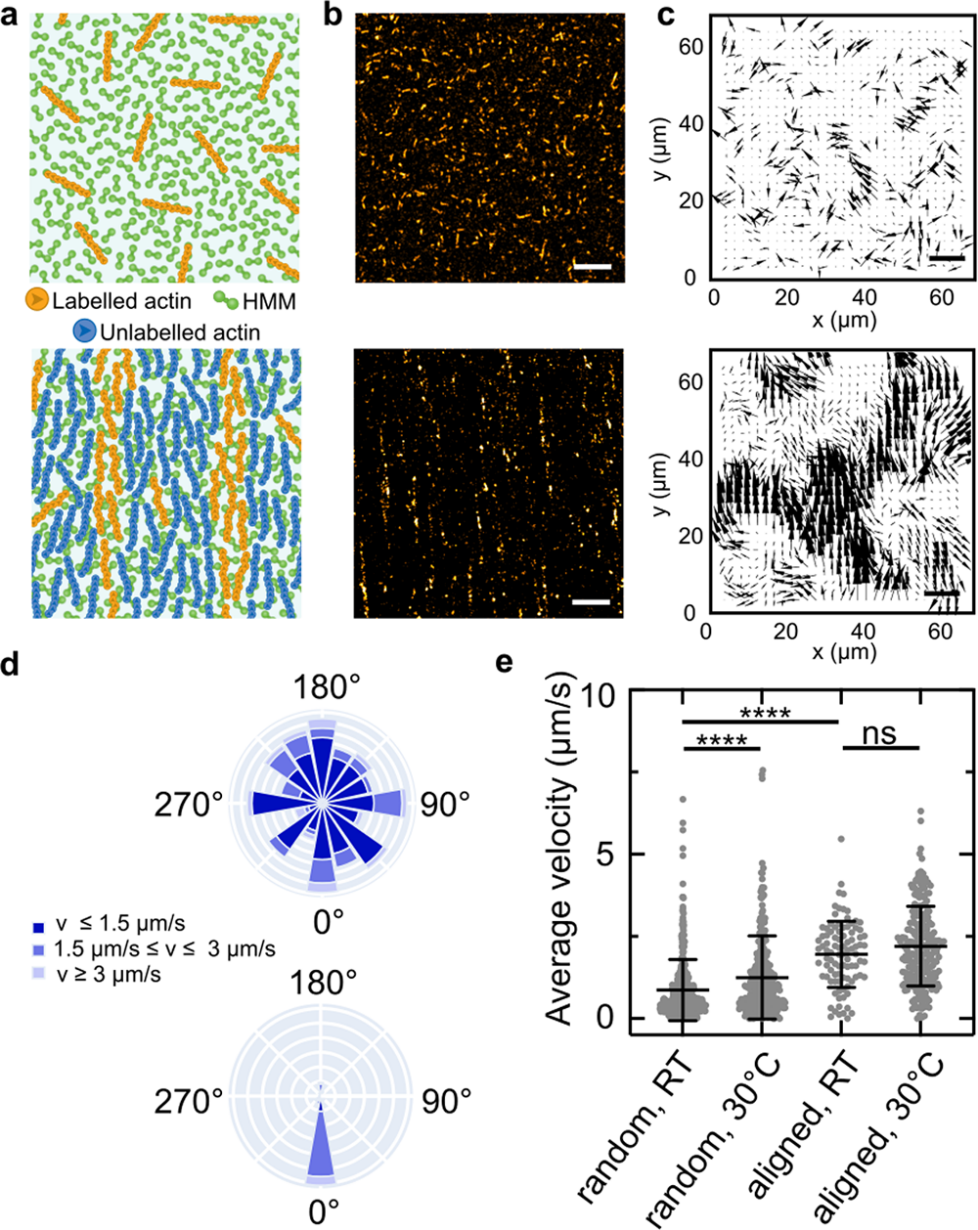
Engineered directional motility of actin filaments. (a) Schematic representations of actin filaments in a standard *in vitro* motility assay moving with random orientations (termed random filaments) or aligned orientations due to nematic ordering at high concentrations (termed aligned filaments). (b) Confocal images of rhodamine-labeled random and aligned actin filaments (λ_ex_ = 561 nm) in an *in vitro* motility assay. (c) Particle image velocimetry of random and aligned actin filaments. (d) Rose diagram depicting the orientation and velocity of random and aligned filaments. In contrast to random filaments, aligned filaments move along one axis with a strong bias (>77%) toward one direction. (e) Actin filament velocity for random and aligned filaments at room temperature and 30 °C. Values depict mean ± SD of *n* ≥ 94 tracked filaments.

Next, we test if we can pull lipid nanotubes from GUVs using the directional force of gliding actin filaments. In order to bind actin filaments to the lipid membrane of GUVs as illustrated in Figure 2a, we prepare filaments with 10% biotinylated actin monomers and verify the successful functionalization with SDS-PAGE (SI Figure S1). Additionally, we form GUVs containing 20 mol % biotinylated lipids. We observe that actin filaments bind to the GUVs in the presence of streptavidin forming an actin exoskeleton on the GUV membrane which links the GUVs to the HMM substrate. A few minutes after addition of the actin-coated GUVs to the *in vitro* motility assay, we observe the formation of lipid nanotubes on the HMM substrate at the bottom of the GUVs (Figure 2b). Note that the GUVs remain intact as proven by the images taken at the equatorial plane (Figure 2b, top). Some GUV clustering is expected due to the use of biotinylated lipids in the presence of streptavidin. We verify the tubular structure of the lipid nanotubes pulled from GUVs using 3D stimulated emission depletion (3D-STED) microscopy (Figure 2c).^30^ The 3D-STED reveals a typical lipid nanotube diameter of around 200 nm (Figure 2d, SI Figure S3). Importantly, the lipid nanotubes are continuously pulled out of the GUVs due to the motor activity, and we can observe the lipid nanotube networks grow over time. Within 37.5 s, more than 30 μm of nanotubes are pulled from a single GUV (Figure 2e). Remarkably, the lipid nanotube networks undergo further remodeling after being pulled from the GUV leading to the emergence of nanotube networks composed of multiple GUVs (SI Video S4). Next, we quantify the network length per GUV for random and aligned actin filaments and GUVs containing 0 or 20 mol % biotinylated lipids (Figure 2f,g). In absence of biotinylated lipids and for random actin filaments, the actin filaments do not bind to the GUVs. Hence, no lipid nanotubes are formed, whereas the network length per GUV and the number of lipid nanotube branches (SI Figure S4) significantly increases in the presence of 20 mol % biotinylated lipids. In accordance with the increased actin filament velocity and directionality, the network length per GUV increases further for aligned actin filaments. Interestingly, even though the trend is the same for random and aligned filaments, we observe pulling of lipid nanotubes even in the absence of biotinylated lipids. We hypothesize that this is due to the high amount of unlabeled F-actin present in the assay to induce the alignment which promotes electrostatic interactions of actin filaments with the GUV membrane (SI Figure S5). This might even be enhanced by the presence of divalent ions in the final buffer.^31^ However, we still observe the longest networks in the presence of 20 mol % biotinylated lipids and for aligned actin filaments. We also find that different lipid compositions can be used to form lipid nanotubes (SI Figure S6). Notably, when we encapsulated a membrane impermeable dye inside the GUV compartment, we find that it can permeate from the GUV lumen into the lipid nanotubes confirming the formation of defect-free hollow nanotubes (SI Figure S7). In summary, it is possible to exploit natural motors to engineer distinct vesicle morphologies which visually resemble neurons.^32^

**Figure 2:**
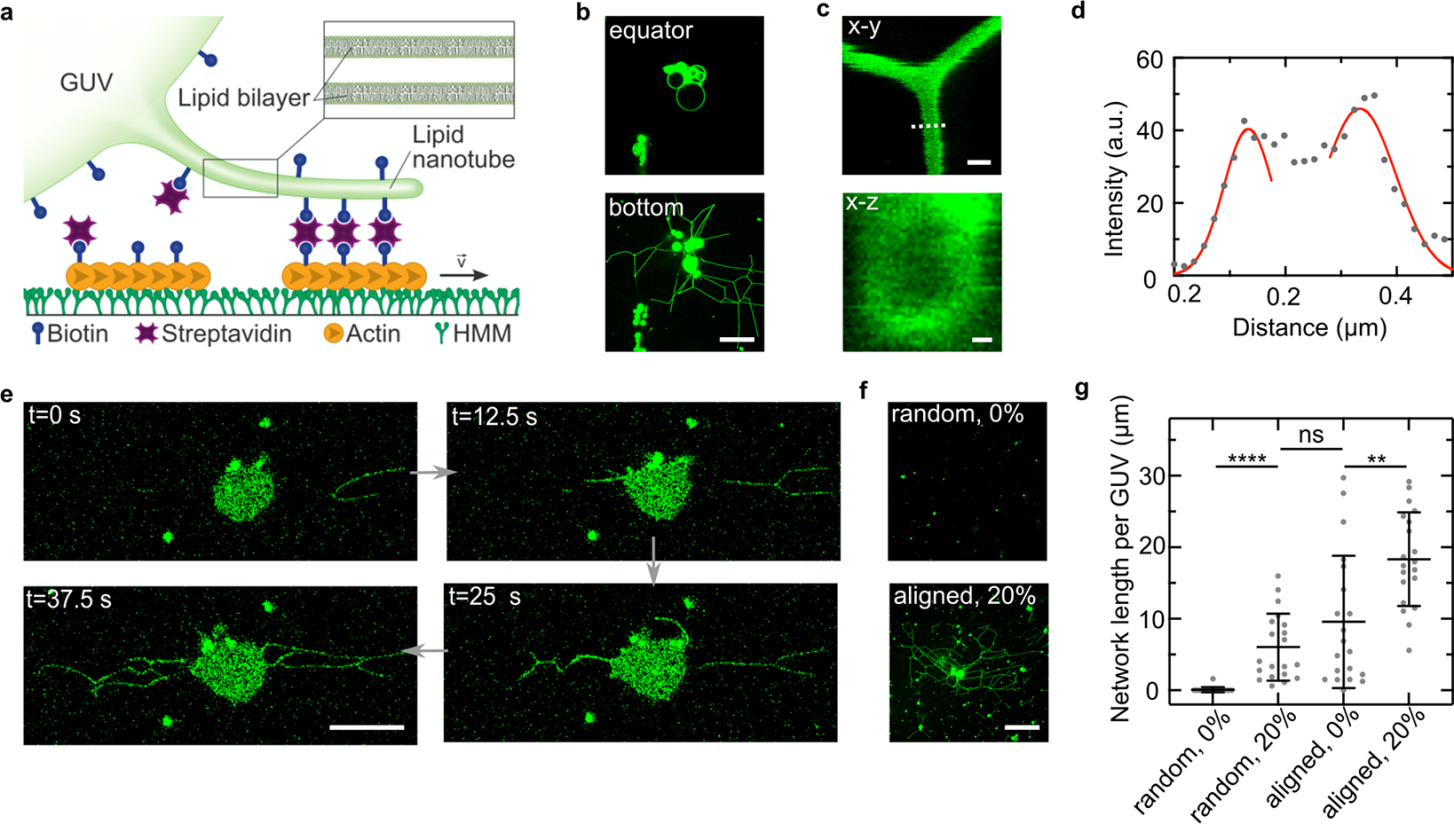
Actin filaments actively pull lipid nanotubes from GUVs. (a) Schematic representation of actin filaments bound to biotinylated lipids in a GUV membrane on an *in vitro* actin motility assay. Motile actin filaments pull lipid nanotubes over the HMM surface. (b) Confocal images of a GUV (membrane labeled with DOPE-488, λ_ex_ = 488 nm) containing biotinylated lipids after pulling of lipid nanotubes in the equatorial (top) and the bottom plane (bottom). Scale bar: 20 μm. (c) 3D-STED images of lipid nanotubes pulled from GUVs. Scale bars: 500 nm (x-y), 100 nm (x-z). (d) Intensity line profile (pixel width: 18 nm) across a lipid nanotube imaged with 3D-STED (indicated as white dashed line in panel c). (e) Confocal time series of a GUV during the lipid nanotube pulling process imaged on the bottom plane at the substrate interface. Scale bar: 20 μm. (f) Confocal images of GUVs in the presence of randomly oriented or aligned actin filaments with 0% or 20% biotinylated lipids, respectively, 60 min after the start of the motility assay. Scale bar: 50 μm. (g) Network length per GUV for randomly oriented or aligned actin filaments with 0% or 20% biotinylated lipids. Values depict mean ± SD for *n* = 20 acquired frames per condition.

As a next step, we translate the nanotube pulling assay from GUVs to living cells, where the membrane is attached to the underlying cytoskeletal cortex. We first probe whether our motor system can pull lipid nanotubes from T-lymphocyte (Jurkat) cells. We verify that cholesterol self-assembles into the cell plasma membrane (SI Figure S8). This allows us to functionalize the Jurkat cells with biotinylated cholesterol. Like for the GUVs, this enables biotinylated actin filaments to bind to the cell in the presence of streptavidin. We observe that the Jurkat cells, despite being nonadherent suspension cells, adhere to the HMM-functionalized substrate due to the artificial linkage established via the actin filaments. Notably, they exhibit many lipid nanotubes at the cell–substrate interface (SI Video S5). By tracking individual cells over time, we find that the pulling of lipid nanotubes mediated by motile actin filaments sets in after about 5 min after the attachment of cells to the HMM (Figure 3a). Note that the cells remain near-stationary during the nanotube pulling process (SI Figure S9 and Videos S6 and S7). Tens of micrometer-long lipid nanotubes are pulled out of each cell over the course of minutes (SI Videos S5, S8, and S9). In the absence of biotinylated cholesterol (0 μM), Jurkat cells do not bind to the substrate and maintain their spherical morphology without the formation of any lipid nanotubes for random as well as aligned filaments (Figure 3b, SI Videos S10 and S11). The absence of unspecific interactions for cells compared to GUVs may be due to their dense glycocalyx, a more complex lipid composition or increased membrane-to-cortex adhesion. By quantifying the network length per cell, we find that whereas the network length for random filaments with 25 μM biotinylated cholesterol is similar to the network length previously determined for GUVs (23 ± 33 μm), the length for aligned filaments exceeds it by an order of magnitude (131 ± 116 μm, Figure 3c). This can partially be explained by the smaller size of the GUVs compared to the Jurkat cells. Since actin-mediated structures provide support of the cell shape and are linked to the cell membrane forming the actin cortex, we focused on the intracellular actin filament organization and dynamics in proximity of lipid nanotubes. Therefore, we stained the intracellular actin with SiR-actin (Figure 3d) and performed live cell imaging. Interestingly, the cellular actin is indeed remodeled and actin filaments are found to extend into the lipid nanotubes, although not along their full length (SI Figure S10). More specifically, we find that no actin filament reaches further than 6 μm into the lipid nanotubes and that they are on average only present within less than half of the nanotube length (SI Figure S11 and Videos S12 and S13). We hypothesize that this is due to the membrane-to-cortex attachment of cellular actin to the cell membrane which causes the actin filaments to be dragged along the membrane during the pulling process. However, the fact that cellular actin filaments only occur at the beginning of nanotubes suggests that the membrane-to-cortex attachment is disrupted at elevated distances and forces of the outer filaments pulling on the cell membrane. Beyond actin, we also stained the mitochondria and the lysosomes of Jurkat cells, as it has been shown that these organelles can be present within tunneling nanotubes of living cells.^23^ However, we do not find any evidence for their presence in the lipid nanotubes pulled from Jurkat cells (SI Figure S12).

**Figure 3:**
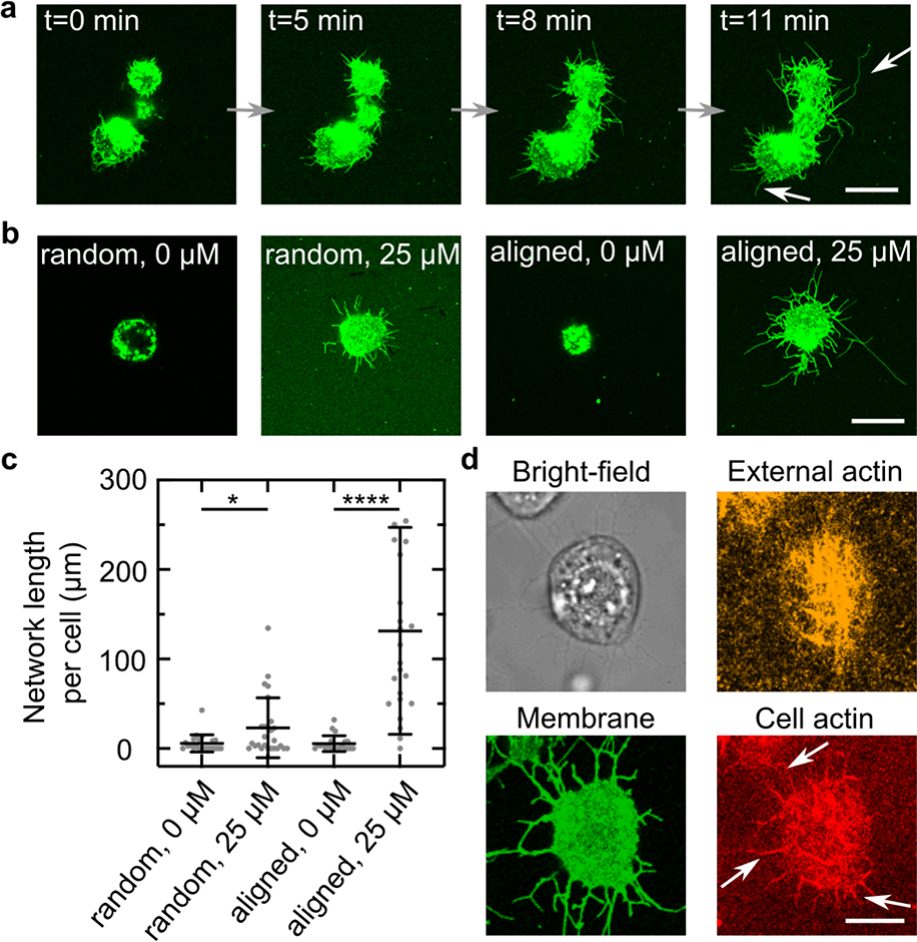
Actin filaments actively pull lipid nanotubes from Jurkat cells. (a) Confocal time series of Jurkat cells (membrane labeled with WGA-Alexa488, λ_ex_ = 488 nm) functionalized with biotinylated cholesterol depicting the pulling of lipid nanotubes over time from the bottom plane at the substrate interface. Scale bar: 20 μm. (b) Confocal images of Jurkat cells in the presence of randomly oriented or aligned actin filaments with 0 or 25 μM biotinylated cholesterol, respectively. Scale bar: 20 μm. (c) Lipid nanotube network length per cell for randomly oriented or aligned actin filaments with 0 or 25 μM biotinylated cholesterol. Values depict mean ± SD of *n* ≥ 21 observed cells for each condition. (d) Confocal live cell images of a Jurkat cell membrane (green, labeled with WGA-Alexa 488, λ_ex_ = 488 nm), extracellular (orange, labeled with rhodamine, λ_*ex*_ = 561 nm), and intracellular actin (red, labeled with SiR-actin λ_*ex*_ = 640 nm). Actin filaments are dragged into lipid nanotubes. Scale bar: 10 μm.

Having shown that molecular motors can pull lipid nanotubes from Jurkat cells, we test if we can expand our assay to a range of different cell types to probe membrane-to-cortex attachment depending on the cells’ adhesive interaction with the surface. We choose keratinocytes (HaCaTs) and fibroblasts (NIH 3T3) as adherent cells and compare them to semiadherent macrophages (J774A.1) and nonadherent Jurkat cells. In order to obtain the best nanotube pulling efficiency, we use aligned actin at 30 °C. As shown in the confocal images in Figure 4a, we observe the pulling of lipid nanotubes for Jurkat cells and macrophages, whereas we do not observe nanotubes for HaCaTs and fibroblasts. We quantify the network length per cell (Figure 4b) and find that Jurkat cells form significantly bigger networks than macrophages (136 ± 116 μm vs 60 ± 58 μm). Noteworthy, the cell size is not the dominant factor that determines the network size per cell as Jurkat cells are smaller than macrophages, keratinocytes, and fibroblasts.^33^ This indicates that the nanotube length is cell type dependent. We hypothesize that no lipid nanotubes form for HaCaTs (SI Figure S13) and fibroblasts due to their high membrane-to-cortex attachment and stiffness compared to nonadherent cells.^3^ The pulling force is therefore likely not sufficient to transiently disrupt the membrane-to-cortex attachment, so that a nanotube can form and actin remodeling can take place. To test this hypothesis, we treat fibroblasts with the actin polymerization inhibitor Latrunculin A (LatA) and perform our lipid nanotube pulling assay. Strikingly, under these conditions we observe the formation of lipid nanotubes (Figure 4c) confirming that the network length per cell is influenced by the membrane-to-cortex attachment of the respective cell type. Importantly, the network length per cell for fibroblasts treated with LatA increased to 312 ± 152 μm and thus exceeds the one for Jurkat cells (136 ± 116 μm, Figure 4d). Possibly, this can be explained by the bigger cell size of fibroblasts or due to the complete absence of any attachment sites for fibroblasts treated with LatA compared to untreated Jurkat cells which still possess membrane-cortex attachment sites.^34^ To summarize, we have shown that our minimal motor-based system can successfully be transferred to natural cells and be used to probe cell type dependent membrane properties.

**Figure 4:**
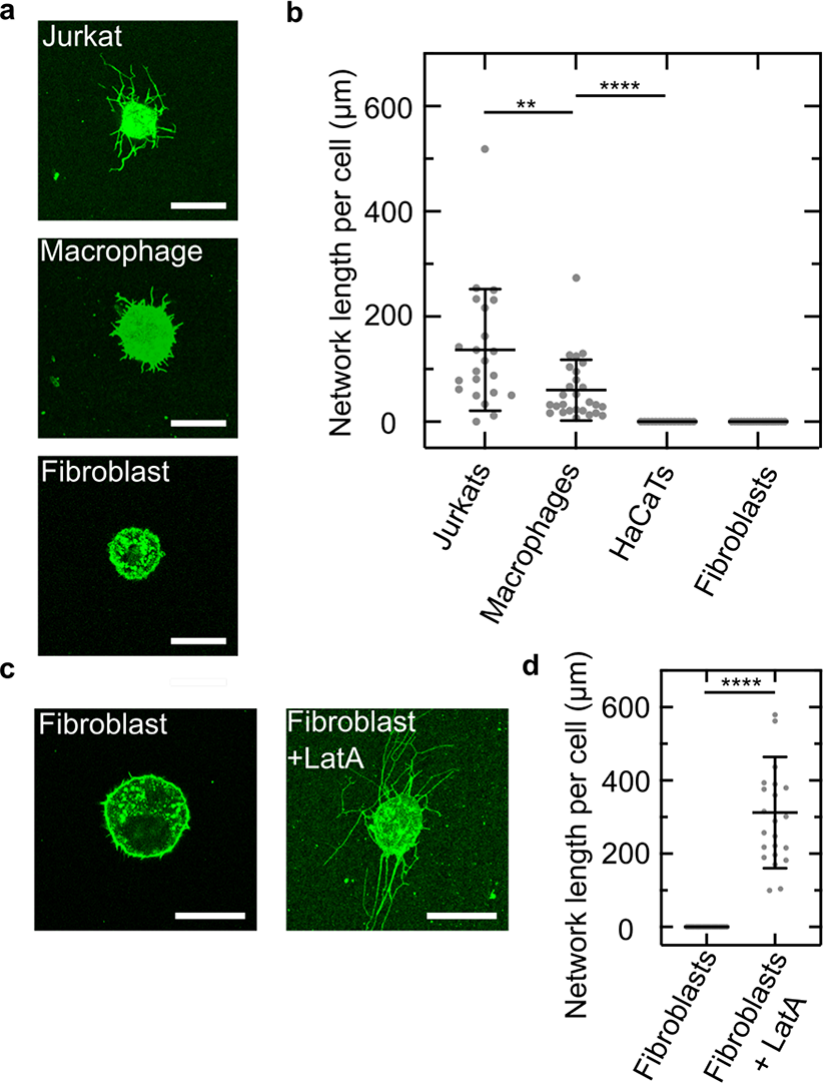
Pulling of lipid nanotubes from different cell types to probe membrane-to-cortex attachment. (a) Confocal images of Jurkat cells, macrophages, and fibroblasts in the presence of 25 μM biotinylated cholesterol and aligned actin filaments on an HMM-coated substrate. Scale bar: 20 μm. (b) Network length per cell for different cell types (mean ± SD of *n* ≥ 15 observed cells for each condition). (c) Confocal images of untreated fibroblasts and fibroblasts treated with the actin-polymerization inhibitor Latrunculin A. Scale bar: 20 μm. (d) Corresponding network length per cell. Inhibiting the actin polymerization allows pulling of lipid nanotubes indicating that this process depends on the cell-to-cortex adhesion. Values depict mean ± SD of *n* ≥ 18 observed cells for each condition.

The question of how mechanical properties of cell membranes and their underlying cortex regulate cell function and behavior is pivotal for a quantitative understanding of force transduction, cell motility, and cell morphology. Here, we developed a minimal system consisting of natural motor proteins that induce membrane deformation and lipid nanotube extraction from GUVs. In the context of bottom-up synthetic biology, this allows one to establish and explore different vesicle morphologies, in particular morphologies that resemble neurons. Moving toward more immediate biological questions, we translate our findings from GUVs to cells, demonstrating how simplified model membrane systems can allow the development of biological assays. By pulling lipid nanotubes from different cell types, we find cell type specific differences in the lipid nanotube length, whereby nonadherent cells exhibit longer nanotubes compared to adherent cells. This pinpoints toward their different membrane mechanics and the level of membrane-to-cortex attachment. Unlike studies using atomic force microscopy or optical tweezers, we are able to screen the mechanical properties on a single-cell level at very high throughput since nanotubes are extracted from multiple cells simultaneously. Moreover, the use of fluorescence microscopy as a readout of cell morphology also allows for the investigation of other cellular processes in real time in parallel. It will be especially exciting to combine this assay with novel fluorescent membrane-tension probes to enhance our understanding of membrane-to-cortex attachment. In general, it will be exciting to witness a conceptual shift from man-made macroscale machines to molecular machines as biophysical tools in the biosciences.

## Supporting Information Available

Additional experimental details and control experiments including further quantification, confocal images and (Experimental Methods 1.1 to 1.18); Supporting Figures S1–S13 with an SDS-PAGE of the purified proteins, a confocal image of actin swirls, 3D-STED images of lipid nanotubes, quantification of the number of lipid nanotube branches pulled from GUVs, images showing unspecific interactions of actin and the GUV membrane, the number of lipid nanotubes pulled from GUVs with different lipid compositions, confocal images of dye permeation into lipid nanotubes of GUVs, the verification of the self-assembly of cholesterol-PEG into cell membranes, confocal images of the GUV and cell displacement over time, confocal images and quantification of cellular actin inside lipid nanotubes, confocal images of stained mitochondria and lysosomes after pulling of lipid nanotubes, and a confocal image of HaCaT cells after the pulling assay (PDF)
Video of time series of random actin filaments (MP4)
Video of time series of aligned actin filaments (MP4)
Video of time series of aligned actin filament patterns (MP4)
Video of time series of lipid nanotube dynamics after pulling from GUVs (MP4)
Video of time series of lipid nanotube pulling from Jurkat cells (MP4)
Video of displacement over time of a Jurkat cell during lipid nanotube pulling (MP4)
Video of displacement over time of a GUV during lipid nanotube pulling (MP4)
Video of 3D projection of Jurkat cells with random actin and biotinylated cholesterol (MP4)
Video of 3D projection of Jurkat cells with aligned actin and biotinylated cholesterol (MP4)
Video of 3D projection of Jurkat cells with random actin and no biotinylated cholesterol (MP4)
Video of 3D projection of Jurkat cells with aligned actin and no biotinylated cholesterol (MP4)
Video of actin filament dynamics during lipid nanotube pulling of a Jurkat cell (DOPE-Atto488) (MP4)
Video of actin filament dynamics during lipid nanotube pulling of a Jurkat cell (SiR-actin) (MP4)
