# Supplementary material for: Actomyosin-Assisted Pulling of Lipid Nanotubes from Lipid Vesicles and Cells
Source: Nano Lett. 2022 Jan 28;22(3):1145–50. doi: 10.1021/acs.nanolett.1c04254 (PMC8832490; doi:10.1021/acs.nanolett.1c04254)
Supplement: Supplementary file 1 — nl1c04254_si_001.pdf [file nl1c04254_si_001.pdf]

Supporting Information: Acto-myosin-assisted pulling of lipid nanotubes from lipid vesicles and cells

Kevin Jahnke[1,2], Stefan J. Maurer[1,2], Cornelia Weber[3], Jochen Estebano Hernandez Bücher[3], Andreas Schoenit[1], Elisa D'Este[4], Elisabetta Ada Cavalcanti-Adam[3], Kerstin Göpfrich[1,2*]

[1]Biophysical Engineering Group, Max Planck Institute for Medical Research, Jahnstraße 29, D-69120 Heidelberg, Germany

[2]Department of Physics and Astronomy, Heidelberg University, D-69120 Heidelberg, Germany

[3]Department of Cellular Biophysics, Max Planck Institute for Medical Research, Jahnstraße 29, D-69120 Heidelberg, Germany

[4]Optical Microscopy Facility, Max Planck Institute for Medical Research, Jahnstraße 29, D-69120 Heidelberg, Germany

*

E-mail: kerstin.goepfrich@mr.mpg.de

Contents

1 Experimental Methods **5**

1.1 Confocal fluorescence microscopy 5

1.2 Actin polymerization 5

1.3 Heavy meromyosin purification 6

1.4 Polyacrylamide gel electrophoresis 6

1.5 *In vitro* motility assay 6

1.6 Particle image velocimetry analysis 7

1.7 Velocity correlation length calculation 8

1.8 Analysis of actin filament velocity 8

1.9 GUV formation 9

1.10 Pulling lipid nanotubes from GUVs 9

1.11 STED microscopy 10

1.12 Analysis of lipid nanotube networks for GUVs 10

1.13 Cell culture 10

1.14 Cell staining 11

1.15 Pulling lipid nanotubes from cells 11

1.16 Analysis of lipid nanotube networks for cells 12

1.17 Statistical analysis 12

1.18 Data availability 13

2 Supporting Figures **14**

Supporting Figure S1: SDS-PAGE of HMM and actin 14

Supporting Figure S2: Aligned actin filaments can form swirls 15

Supporting Figure S3: 2D- and 3D-STED reveals the tubular structures of lipid nanotubes 16

Supporting Figure S4: Total number of lipid nanotube branches pulled from GUVs 17

Supporting Figure S5: Unspecific interaction of actin and DOPC bilayers in presence of divalent ions 18
Supporting Figure S6: Nanotube networks for different lipid compositions 19
Supporting Figure S7: Encapsulated dye permeates into lipid nanotubes of GUVs 20
Supporting Figure S8: Self-assembly of cholesterol-PEG into membranes of different cell types 21
Supporting Figure S9: GUVs and cells show little to no movement during the lipid nanotube pulling assay 22
Supporting Figure S10: Actin filaments are present within lipid nanotubes pulled from cells 23
Supporting Figure S11: Characterization of actin filaments extending into lipid nanotubes 24
Supporting Figure S12: Mitochondria and lysosomes do not enter lipid nanotubes of Jurkat cells 25
Supporting Figure S13: HaCaT cells do not form lipid nanotubes 26

3 Supporting Videos 27

Supporting Video S1: Time series of random actin filaments 27
Supporting Video S2: Time series of aligned actin filaments 27
Supporting Video S3: Time series of aligned actin filament patterns 27
Supporting Video S4: Time series of lipid nanotube dynamics after pulling from GUVs 27
Supporting Video S5: Time series of lipid nanotube pulling from Jurkat cells . . . 28
Supporting Video S6: Displacement over time of a Jurkat cell during lipid nanotube pulling 28
Supporting Video S7: Displacement over time of a GUV during lipid nanotube pulling 28

Supporting Video S8: 3D projection of Jurkat cells with random actin and biotinylated cholesterol 28
Supporting Video S9: 3D projection of Jurkat cells with aligned actin and biotinylated cholesterol 29
Supporting Video S10: 3D projection of Jurkat cells with random actin and no biotinylated cholesterol 29
Supporting Video S11: 3D projection of Jurkat cells with aligned actin and no biotinylated cholesterol 29
Supporting Video S12: Actin filament dynamics during lipid nanotube pulling of a Jurkat cell (DOPE-Atto488) 29
Supporting Video S13: Actin filament dynamics during lipid nanotube pulling of a Jurkat cell (SiR-actin) 30

References **30**

1 Experimental Methods

1.1 Confocal fluorescence microscopy

A confocal laser scanning microscope LSM 880 or LSM 900 (Carl Zeiss AG) was used for confocal imaging. The pinhole aperture was set to one Airy Unit and the experiments were performed at room temperature. The images were acquired at RT or 30 °C using a 63× oil immersion objective (Plan-Apochromat 63×/1.4 Oil DIC M27). Images were analyzed and processed with ImageJ (NIH, brightness and contrast adjusted).

1.2 Actin polymerization

Actin from New Zealand white rabbit skeletal muscle was purified from acetone powder based on the method of Pardee and Spudich,[1] modified after Kron *et al.*[2] To form actin filaments, we mixed the actin monomers 1/10 with the standard working buffer named 10× Actin Buffer (AB, containing 250 mM imidazol-HCl, 250 mM KCl, 10 mM EGTA, 40 mM $MgCl_2$, pH 7.4) and 1/10 APB 10× Actin Polymerization Buffer (APB, 20 mM Tris-HCl, pH 8, 500 mM KCl, 20 mM $MgCl_2$, 10 mM Na_2ATP), to make a 20 µM actin stock. The actin/APB mix was left at RT to polymerize for 20-30 min. Subsequently, 8 units of rhodamine-phalloidin (from Invitrogen dissolved in MeOH) were added to stabilize the actin filaments after evaporating half the volume of methanol. Biotinylated actin bFa: G-actin monomers were mixed in a ratio of 10:1 with biotinylated G-actin (Cytoskeleton, Cat #AB07) and left to polymerize as described before after addition of 10 µL vaporized rhodamin-phalloidin (8 units) to the 20 µL actin mix. Biotinylated actin was aliquoted and stored at −80 °C until use. Unlabeled F-actin FA: G-actin monomers (5 mg/ml) were mixed in AB_{DTT} buffer (standard working buffer supplemented with 20 mM DTT) and 1/10 of APB. The solution was left to polymerize for 30 min at RT and then kept on ice. Directly before use, the solution was diluted to 1 mg/mL in AB_{GOC} (AB supplemented with 3 mg/ml glucose, 0.1 mg/mL glucose oxidase, 0.02 mg/mL catalase and 20 mM DTT).

1.3 Heavy meromyosin purification

Myosin isolated from New Zealand white rabbit skeletal muscle was used to prepare HMM based on the method of Margossian and Lowey.[3] To obtain a highly functional motor driven motility, we first remove non-functional myosin heads (rigor heads) *via* an actin affinity purification.[4] In short, the myosin motor fragments (heavy meromyosin, HMM) are mixed with actin filaments and MgATP in solution, followed by ultracentrifugation at 1×10^5 g to pellet any MgATP insensitive motors together with the actin filaments. The high functional HMM then is supplemented with 20 % sucrose and will be stored at −80 °C until use.

1.4 Polyacrylamide gel electrophoresis

Samples of protein were electrophoresed on a precast gradient SDS-PAGE of 4-12 % bis-tris gel (1 mm, NuPage, Invitrogen) according to the Invitrogen protocol. For HMM 5 µg protein were loaded per lane and for actin 1-7 µg protein. The gel was run for 50 min at 200 V. After the electrophoresis the gel was stained for 30 min with the colloidal Comassie stain ReadyBlue (Sigma-Aldrich). Imaging was performed with the Azure 600 (Azure Biosystems).

1.5 *In vitro* motility assay

A thin glass slide (24 x 60 mm^2, Menzel) was washed with isopropanol in an ultrasonic bath and dried afterwards. Subsequently, they were dip-coated with 0.3 % nitrocellulose solved in amylacetate and allowed to dry overnight. A second coverslip (18 x 18 mm^2, Menzel) was placed on the top of the coated glass slide spaced with two stripes of a double-sided sticky tape (tesa SE, Germany) to form a flow cell chamber FC with 0.1 mm gap and 15 µL volume. The standard working solution (called AB) was prepared as a 10x concentrated stock solution made of (250 mM imidazole-HCl, 250 mM KCl, 10 mM EGTA, 40 mM $MgCl_2$, pH 7.4). Aliquots of 1 ml were stored in −20 °C. On the day of the experiment the 10x AB stock solution was thawed and the respective supplements were added. We used double

concentrated AB solutions to match the osmotic conditions of GUVs. To minimize the contamination with atmospheric oxygen the mixed solutions were degassed in a vacuum exicator for at least 20 min in the cold room. HMM solution (200 µg L^{-1}) was infused and immobilized in the flow cell. After two minutes the unbound HMM was washed off with 2x AB containing 20 mM dithiothreitol (DTT, AB_{DTT}) and then the liquid was exchanged by 2x AB supplemented with bovine serume albumin (BSA) (0.5 mg/mL, AB_{BSA}). The BSA is incubated for two minutes to block binding of labeled actin to the nitrocellulose surface of the coverslip. The flow cell was then washed with 2x AB_{DTT}. For aligned actin filaments, F-actin solution (1 mg/mL in AB_{GOC}) was washed in the flow cell for another two minutes. Unbound F-actin was removed with 2x AB_{DTT} followed by a wash with 2x AB_{GOC} (AB supplemented with 3 mg/ml glucose, 0.1 mg/ml glucose oxidase, 0.02 mg/ml catalase, 20 mM DTT). GOC substances prevent oxidative stress and photobleaching. The ATP-containing solution termed AB_{MC} was supplemented with 5 mM MgATP, GOC, DTT and 0.3 % methylcellulose (MC). MC was purchased from Sigma Aldrich (4000 cP viscosity at 2 % w/v and a molecular weight of about 500 kDa. Finally, the flow cell was flushed with the GUV or the cell mixture in the respective experiments.

1.6 Particle image velocimetry analysis

The background of timelapse videos was subtracted. Therefore, the minimal intensity of the stack was projected and then subtracted using the Calculator Plus plugin in Fiji. Furthermore, images were rotated in a way that actin filaments move from left to right. The individual images of the stack were loaded into JPIV (https://eguvep.github.io/jpiv/index.html) run in a Python environment. To obtain the vector field, particle image velocimetry was performed on consecutive images using first a 64x64 and then a 16x16 pixel interrogation window with a final vector spacing of 8x8 pixel. The vector fields were batch-filtered by performing a normalized median test and a median filter, where all invalid vectors were excluded. These invalid vectors were replaced by the median to obtain the final vector field.

1.7 Velocity correlation length calculation

A custom-written Python script was used to format the JPIV data for further processing. The velocity correlation length of the actin fibers was calculated in MATLAB using a script described elsewhere.[5,6] In brief, the displacement vectors were divided by the time difference between the two images from which they were generated, resulting in the velocity vector $\mathbf{r}_{i,j}$, which was assigned to the central coordinate (i,j) of each 8x8 window. Since the axial movement is the dominant direction in the described experimental setup, only the lateral component $\mathbf{U}_{i,j}$, perpendicular to the movement direction was used to calculate the velocity fluctuations $\mathbf{u}_{i,j}$ as:

$$\mathbf{u}_{i,j} = \mathbf{U}_{i,j} - \sum_{i=1,m} \sum_{j=1,n} \frac{\mathbf{U}_{i,j}}{m \times n} = \mathbf{U}_{i,j} - \mathbf{U}_{\text{mean}}$$

$\mathbf{U}_{\text{mean}}$ is the mean velocity. The lateral correlation function $\mathbf{C}_r$ was calculated as:

$$\mathbf{C}_r = \frac{\langle \mathbf{u}(r') * \mathbf{u}(r'+r) \rangle_{r'}}{\sqrt{\langle \mathbf{u}(r')^2 \rangle * \langle \mathbf{u}(r'+r)^2 \rangle}}$$

$\langle \ldots \rangle$ is the average and $r = \|\mathbf{r}_{i,j}\|$ is the norm of $\mathbf{r}_{i,j}$. The first crossing of the threshold 0.01 with the lateral correlation function $\mathbf{C}_r$ was defined as the velocity correlation length.

1.8 Analysis of actin filament velocity

Moving actin filaments were recorded as a time-lapse and tracked using ImageJ plugins: A classifier in the Trainable Weka Segmentation plugin[7] was trained to detect filaments and create a binary map of well defined particles against the background. This improves the ability of the Trackmate plugin[8] to correctly identify, track and return the trajectories of the individual particles. For each trajectory the magnitude of the average velocity vector and the orientation, *i.e.* the argument of the end-to-end vector were calculated. To display the data in a rose plot the trajectories were binned first by their argument and then within

these bins by the magnitude of their average velocity vector. The rose plots were generated using the plotly library (v4.14.3) for python (v3.7.4).

1.9 GUV formation

Giant unilamellar vesicles were prepared using the electroformation method[9] using a VesiclePrepPro device (Nanion Technologies GmbH). 40 µL of 5 mM lipid mix (containing 99 % 1,2-dioleoyl-sn-glycero-3-phosphocholine (DOPC) and 1 % 1,2-dioleoyl-sn-glycero-3-phospho ethanolamine-Atto488 (Atto488-PE)) in $CHCl_3$ were homogeneously spread on the conductive side of an indium tin oxide (ITO) coated glass slide (Visiontek Systems Ltd). After evaporating the chloroform for 20 min under vacuum, a rubber ring was placed on the lipid-coated ITO slide and filled with 275 µL of 300 mM sucrose solution to match the omsolarity of the double-concentrated AB buffer. The second ITO slide was put on top and the chamber connected to the electrodes of the VesiclePrepPro. An AC field (3 V, 5 Hz) was applied *via* the electrodes for 138 min while the solution was heated to 37 °C. GUVs were collected immediately after electroformation and stored at 4 °C for up to 7 days.

1.10 Pulling lipid nanotubes from GUVs

In order to pull lipid nanotubes from GUVs, 10 µL biotinylated GUVs were incubated for 1 min with 2 µL streptavidin (final concentration 90 nM, Sigma Aldrich) and 0.4 µL biotinylated F-actin filaments (bFa*, final concentration 20 nM). Subsequently, they were mixed with 3.1 µL methylcellulose (final concentration 0.31 w/v%), 1 µL MgATP (final concentration 5 mM) and 3.5 µL AB_{MC}. Afterwards the solution was flushed immediately into the flow cell, which was sealed with two-component glue.

1.11 STED microscopy

Lipid nanotubes were imaged on an Abberior expert line (Abberior Instruments GmbH, Germany) with a pulsed STED line at 775 nm using an excitation laser at 640 nm and spectral detection. The detection window was set between 650–750 nm to detect Atto633-labeled lipid nanotubes. Images were acquired with a $100\times/1.4$ NA magnification oil immersion lens (Olympus). The pixel size was set to 15-18 nm and the pinhole was set to 0.8 AU for 2D-STED and to 0.6 AU for 3D-STED. Images were analyzed and processed with ImageJ (NIH, brightness and contrast adjusted).

1.12 Analysis of lipid nanotube networks for GUVs

GUVs and nanotubes were classified separately using two different classifiers in the trainable Weka Segmentation plugin. In the segmented image the individual GUVs (imaged cross-sectional area larger than 6 μm^2) are then counted and the nanotubes skeletonized, *i.e.* reduced to one-dimensional branches, whose individual lengths can be determined using the Analyze Skeleton plugin. For each micrograph, the sum of all branch lengths, *i.e.* the network length is calculated, omitting branches smaller than 10 µm to exclude artefacts. The network length is divided by the respective number of GUVs to obtain the normalized network length per GUV for one micrograph, from which the average and standard deviation displayed in the text were calculated.

1.13 Cell culture

Jurkat cells (clone E6.1) were cultured in suspension in Roswell Park Memorial Institute 1640 Medium supplemented with GlutaMax^{TM}, (RPMI 1640, ThermoFisher Scientific) 1 % penicillin/streptomycin (ThermoFisher Scientific) and 10 % fetal bovine serum (Sigma Aldrich) at 37 °C and 5 % CO_2 atmosphere. Cells were passaged by transferring 10 ml of cell suspension to 30 ml of fresh cell culture medium every 2-3 days. HaCaT and NIH3T3 cells were cul-

tured in Dulbecco‘s Modified Eagle Medium supplemented with GlutaMax™ (DMEM, ThermoFisher Scientific), 1 % penicillin/streptomycin and 10 % fetal bovine serum or 10 % FBS South American HI (ThermoFisher Scientific) at 37 °C and 5 % CO_2 atmosphere and passaged at approximately 80 % confluency by 0.05 % trypsin/EDTA treatment. J774A.1 macrophages were cultured in Dulbecco‘s Modified Eagle Medium supplemented with GlutaMax™ and 10 % fetal bovine serum at 37 °C and 5 % CO_2 atmosphere. Subcultures were prepared by scraping and transferring 10 ml of cell suspension to 30 ml of fresh cell culture medium every 3-4 days. Approximately 250 000 cells/ml were used for the experiments.

1.14 Cell staining

For the staining of the plasma membrane Wheat Germ Agglutinin conjugates (Thermo Fisher Scientific) were added at a final concentration of 10 µg/ml to the respective cells. In order to stain the actin filament a final concentration of 10 µM verapamil and 1 µM SiR-Actin (SpiroChrome) was added to the cells. Nih3T3 fibroblasts were treated with a final concentration of 0.2 µg mL^{-1} Latrunculin A (Enzo Life Sciences) and incubated for 1 h. For the co-culture experiments Jurkat cells were either stained with CellTracker blue CMHC™ (ThermoFisher Scientific), at final concentration of 20 µg/ml, or Wheat Germ Agglutinin Alexa Fluor™ 647 conjugate as described above. In order to evaluate the cell viability propidium iodide (ThermoFisher Scientific) was added at a final concentration of 1.5 ng/ml to the Jurkat cell suspension. Lysosomes were stained using LysoTracker Green DND-26 (ThermoFisher Scientific) at a final concentration of 75 nM. For the staining of mitochondria MitoTracker FM green (ThermoFisher Scientific) was added at a final concentration of 500 nM to the cells.

1.15 Pulling lipid nanotubes from cells

Lipid nanotubes were pulled from cells at comparable cell densities around 1×10^6 cell/ml. 98.5 µL of the cell suspension in medium was incubated for 10 min with 1 µL cholesterol-

PEG-biotin (10 mM, Nanocs) and 0.5 µL membrane staining wheat germ agglutinin conjugated with Alexa488 (WGA-Alexa488). After the incubation, 5 µL of the suspension was mixed with 2 µL bFa* (final concentration 100 nM), 1 µL streptavidin (final concentration 910 nM), 1 µL MgATP (final concentration 5 mM), 3.1 µL methylcellulose (final concentration 0.31 s/v% and 7.9 µL AB_{MC}. Afterwards the solution was flushed immediately into the flow cell, which was sealed with two-component glue.
To test the self-assembly of cholesterol-PEG into the cell membrane, we incubated 99 µL of the cell suspension for 10 min with 1 µL Chol-PEG-FITC (10 mM, Nanocs).

1.16 Analysis of lipid nanotube networks for cells

Micrographs containing several cells were cropped, so that one image contains only a single cell. The trainable Weka Segmentation classifier was trained to separate protrusions extending into the periphery of the cell from the cell itself and the background. For each cell type, an individual classifier was trained. Again, the branch lengths of the lipid nanotubes were determined by skeletonization. For the branches a cut-off of 2.5 µm was chosen to only consider nanotubes and no natural cell protrusions like lamellipodia. For each cell the network length was determined and the averages and standard deviations were calculated for each cell type.

1.17 Statistical analysis

All the experimental data is reported as mean ± SD from n experiments. The respective value for n is stated in the corresponding figure captions. All experiments were repeated at least twice. To analyze the significance of the data, a Student's t-test with Welch's correction was performed using Prism GraphPad (Version 9.1.2) and p-values correspond to ****: $p \leq 0.0001$, ***: $p \leq 0.001$, **: $p \leq 0.01$, *: $p \leq 0.05$ and ns: $p \geq 0.05$.

1.18 Data availability

The datasets generated during and analyzed during the current study are available from the corresponding author on reasonable request.

2 Supporting Figures

Supporting Figure S1: SDS-PAGE of HMM and actin

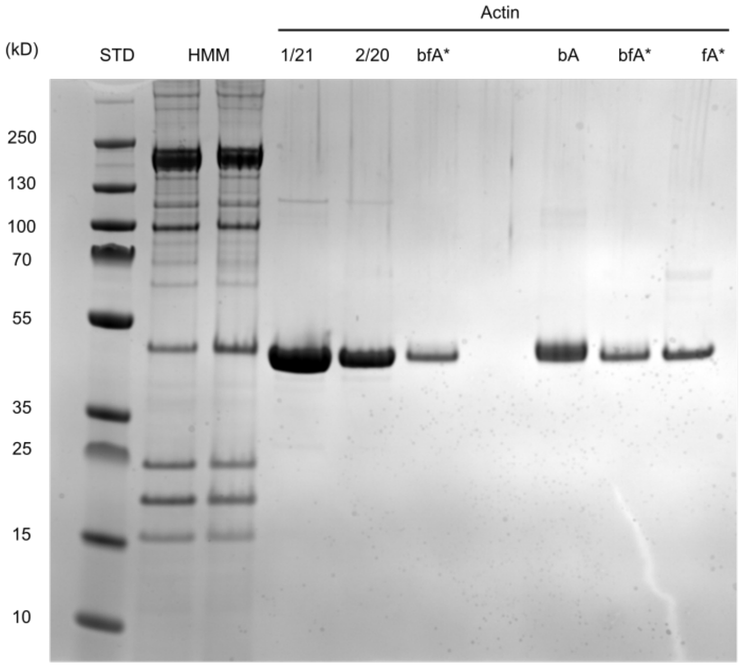

Figure S1: Denaturing polyacrylamide gel electrophoresis (SDS-PAGE) of HMM and actin after purification. HMM: The strong band at 225 kDa indicates the successful HMM purification including the light chains between 15 and 25 kDa. The actin band at 42 kDa results from remnants of the actin affinity purification. Actin: All actin samples show a strong band at 42 kDa. These include two batches of purified actin (1/21 and 2/20) used for this study, polymerized biotinylated actin filaments (bfA*), pure biotinylated actin monomers (bA) and polymerized actin filaments without biotin (fA*).

Supporting Figure S2: Aligned actin filaments can form swirls

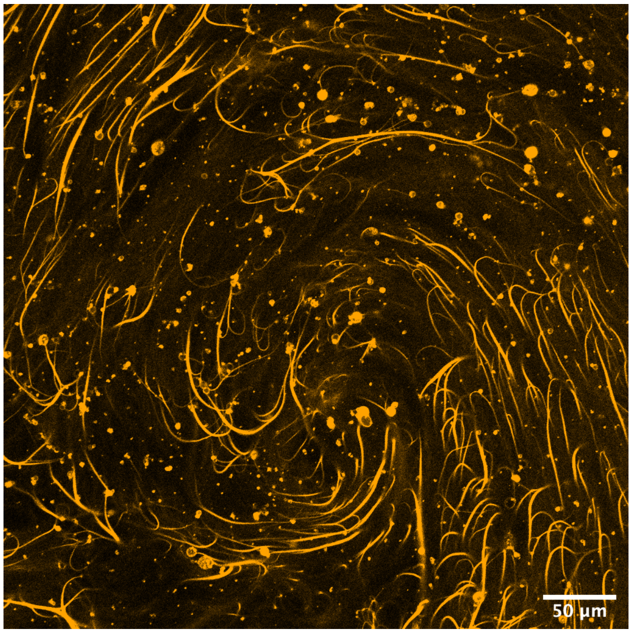

Figure S2: Aligned actin filaments can form swirls. Confocal image of aligned actin filaments (labeled with Rhodamine B, $\lambda_{ex} = 561\,\text{nm}$) after 1 h of incubation. Occasionally, aligned actin filaments form swirl-like patterns such that the global direction of the motion is changing.[10] Scale bar: 50 µm.

Supporting Figure S3: 2D- and 3D-STED reveals the tubular structures of lipid nanotubes

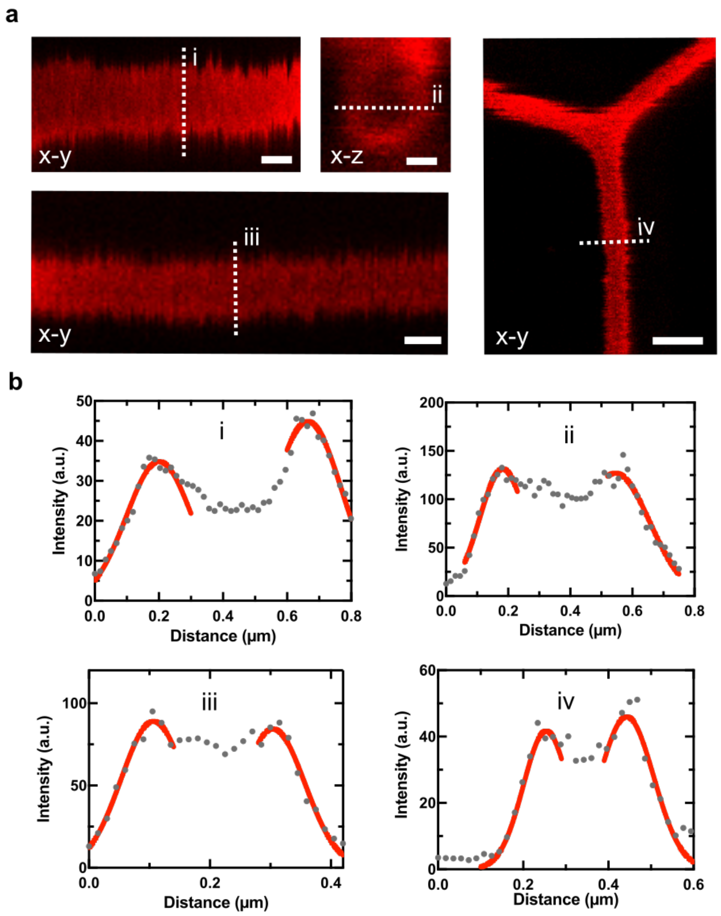

Figure S3: 2D- and 3D-STED reveals the tubular structures of lipid nanotubes. **a** 2D- and 3D-STED images of lipid nanotubes pulled from GUVs (membrane labeled with DOPE-Atto633, $\lambda_{ex} = 640\,\text{nm}$) depict the hollow tubular structure of lipid nanotubes. Scale bars: i, ii, iii - 200 nm, iv - 500 nm. **b** Line profiles (pixel width: 18 nm) across lipid nanotubes as indicated in **a**. The intensity profiles were fitted with gaussian fits at the position of the bilayer, which reveal lipid nanotube diameters of 463 nm (i), 362 nm (ii), 197 nm (iii) and 189 nm (iv), which were calculated as the difference of the position of the gaussian fit maxima.

Supporting Figure S4: Total number of lipid nanotube branches pulled from GUVs

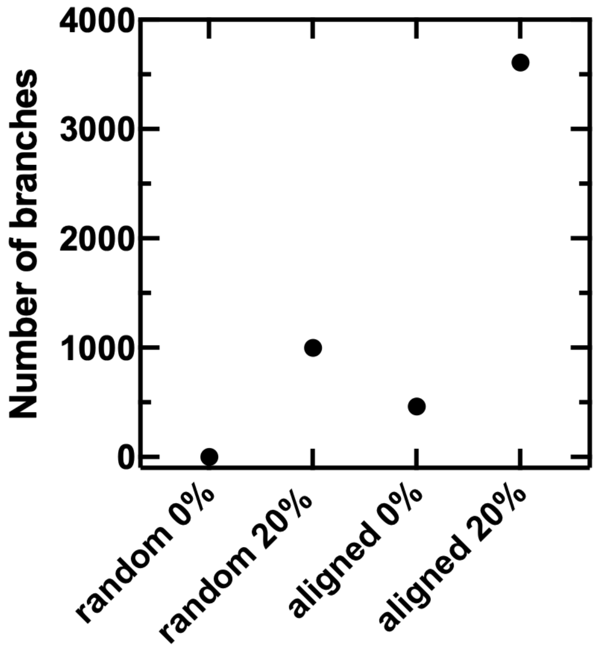

Figure S4: Total number of lipid nanotube branches pulled from GUVs. The number of branches (edge connecting two nodes of a network) increases with the presence of 20 % biotinylated lipids and is generally higher for aligned actin filaments. The high amount of branches for 0 % biotinylated lipids and aligned filaments can be attributed to the high amount of unlabelled F-actin present in the assay to induce the alignment which promotes unspecific interactions of actin filaments with the GUV membrane. This may be enhanced by the presence of divalent ions in the final buffer.[11]

Supporting Figure S5: Unspecific interaction of actin and DOPC bilayers in presence of divalent ions

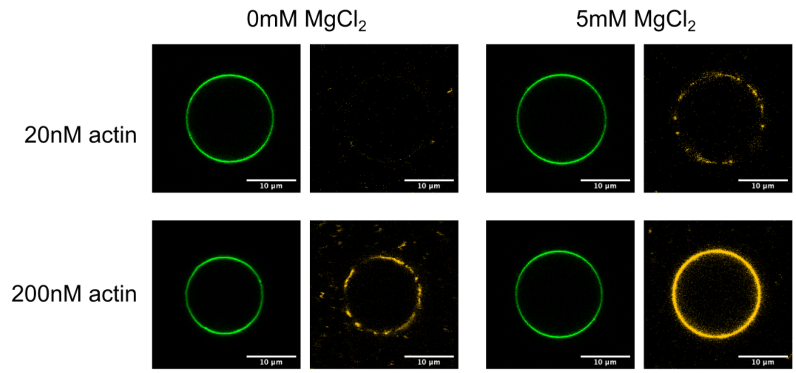

Figure S5: Unspecific interaction of actin and DOPC bilayers in presence of divalent ions. Confocal images of giant unilamellar vesicles (green, membrane labeled with DOPE-488, $\lambda_{ex} = 488\,\mathrm{nm}$) in presence of 0 or 5 mM $MgCl_2$ and 20 or 200 nM filamentous actin (orange, labeled with rhodamine, $\lambda_{ex} = 561\,\mathrm{nm}$), respectively. As visible in the confocal images divalent ions increase the amount of unspecific actin filament binding to the GUV membrane. Additionally, high actin concentrations lead to interactions with the membrane. This explains the presence of lipid nanotubes for aligned actin filaments and GUVs containing 0 % biotinylated lipids. Scale bar: 10 µm.

Supporting Figure S6: Nanotube networks for different lipid compositions

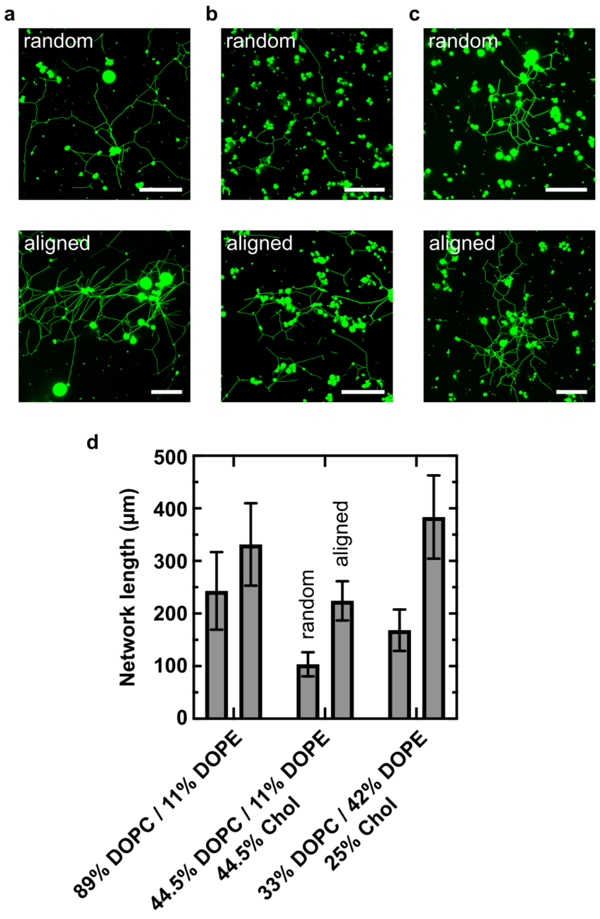

Figure S6: Nanotube networks can be achieved for biotinylated GUVs with different lipid compositions for random and aligned actin filaments. **a** 89 % DOPC, 11 % DOPE; **b** 44.5 % DOPC, 11 % DOPE, 44.5 % cholesterol; **c** 33 % DOPC, 42 % DOPE, 25 % cholesterol. Scale bar: 40 µm. **d** Mean network length (sum of all branch lengths belonging to one network) with standard error of mean for different lipid compositions. For all conditions, the network length is on average larger when aligned actin filaments (right bar, respectively) are used compared to random filaments (left bar, respectively). Furthermore, the lipid composition seems to influence the network length, whereby increased cholesterol content leads to a decrease in the network length.

Supporting Figure S7: Encapsulated dye permeates into lipid nanotubes of GUVs

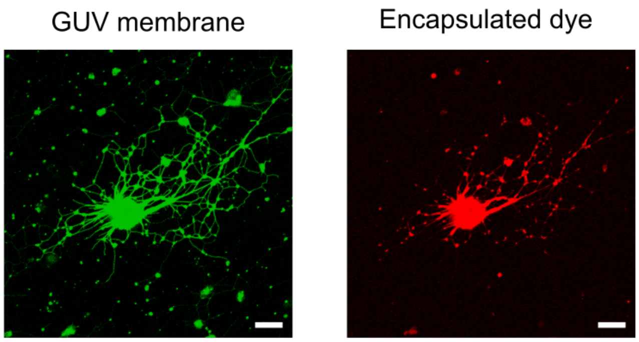

Figure S7: Encapsulated dye permeates into lipid nanotubes of GUVs. Confocal images of a GUV (membrane labeled with Atto488-DOPE, λ_{ex} =488 nm) with an encapsulated dye (Alexa Fluor 647-NHS ester, λ_{ex} =640 nm). After nanotube formation the encapsulated dye is also visible within the lipid nanotubes. Scale bar: 20 µm.

Supporting Figure S8: Self-assembly of cholesterol-PEG into membranes of different cell types

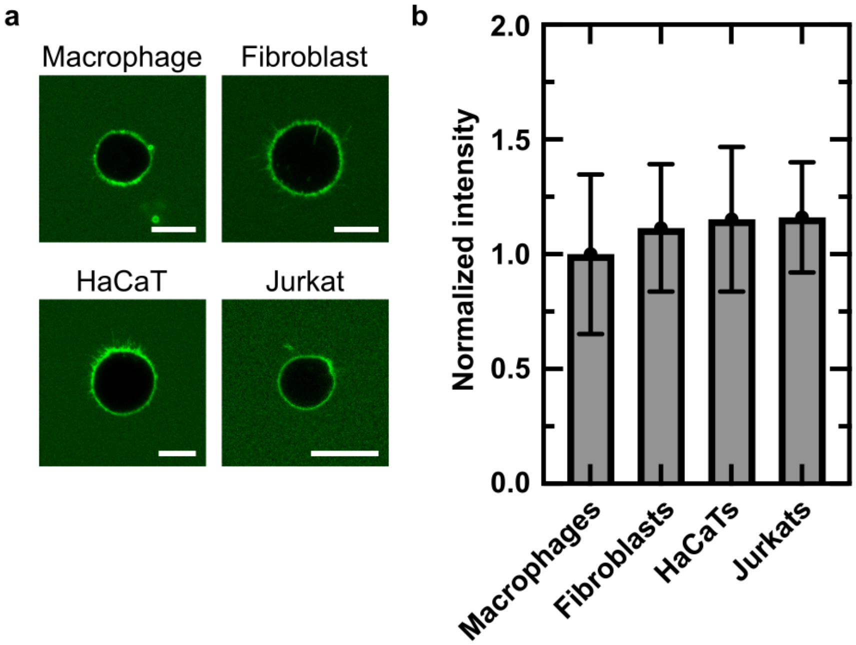

Figure S8: Self-assembly of cholesterol-PEG into membranes of different cell types. Cholesterol-PEG-FITC inserts equally well into the membranes of all cell types used in this study. This verifies the successful functionalization with biotinylated cholesterol. **a** Confocal images of the equatorial plane of the different cell types as indicated. Scale bar: 10 µm. **b** Average intensities of the fluorescence captured from the equatorial plane of each individual cell type (n $\geq$ 16), normalized to the minimal fluorescence intensity.

Supporting Figure S9: GUVs and cells show little to no movement during the lipid nanotube pulling assay

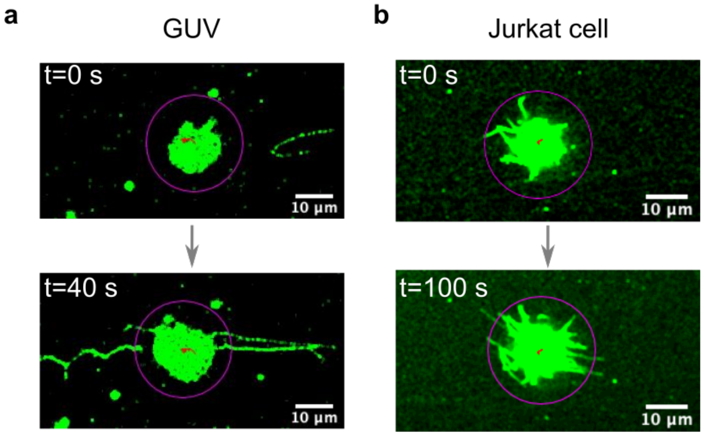

Figure S9: GUVs and cells show little to no movement during the lipid nanotube pulling assay. GUV (**a**, membrane labeled with DOPE-488, $\lambda_{ex} = 488\,\text{nm}$)) and Jurkat cell (**b**, membrane labeled with WGA-Alexa488, $\lambda_{ex} = 488\,\text{nm}$) during actom-myosin assisted lipid nanotube formation. Over the time course of seconds to minutes there little to no movement with an mean squared displacement of 2.9 µm or 1.3 µm of the lipid vesicles or cell center, respectively, during nanotube formation (network length >100 µm and >40 µm, respectively). The trajectories of the lipid vesicle and the Jurkat cell (violet circle) were tracked with ImageJ (Trackmate[12]) and are shown as red lines in the confocal images. Scale bar: 10 µm.

Supporting Figure S10: Actin filaments are present within lipid nanotubes pulled from cells

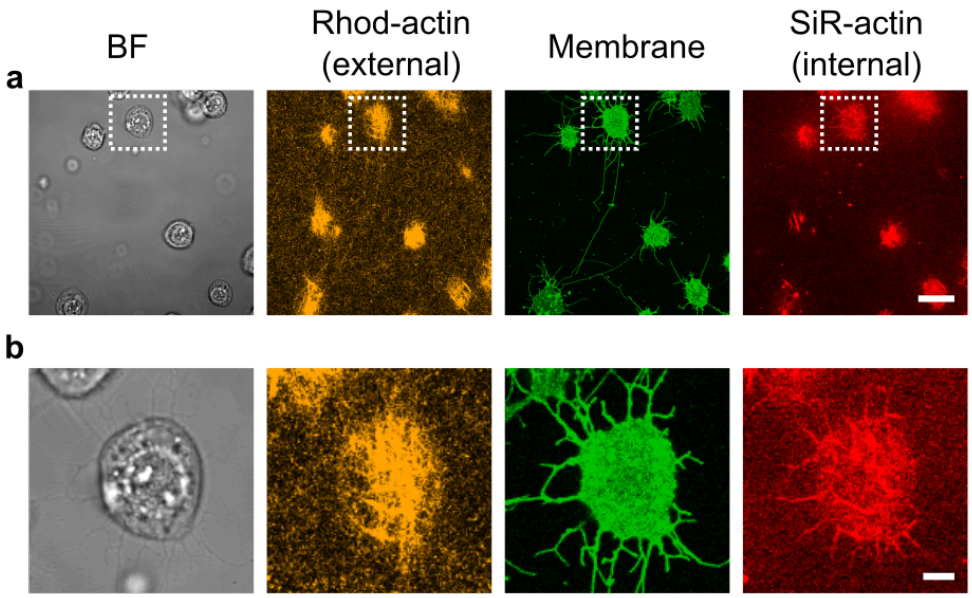

Figure S10: Actin filaments are present within lipid nanotubes pulled from cells. **a,b** Overview (**a**) and zoomed (**b**) confocal fluorescence images of Jurkat cells (membrane labeled with WGA-Alexa488, $\lambda_{ex} = 488\,\text{nm}$), extracellular actin filaments (labeled with Rhodamine $\lambda_{ex} = 561\,\text{nm}$) and intracellular actin filaments (labeled with Silicon-Rhodamine Actin $\lambda_{ex} = 640\,\text{nm}$). Actin filaments are dragged into the lipid nanotubes. Scale bars: 20 µm and 5 µm.

Supporting Figure S11: Characterization of actin filaments extending into lipid nanotubes

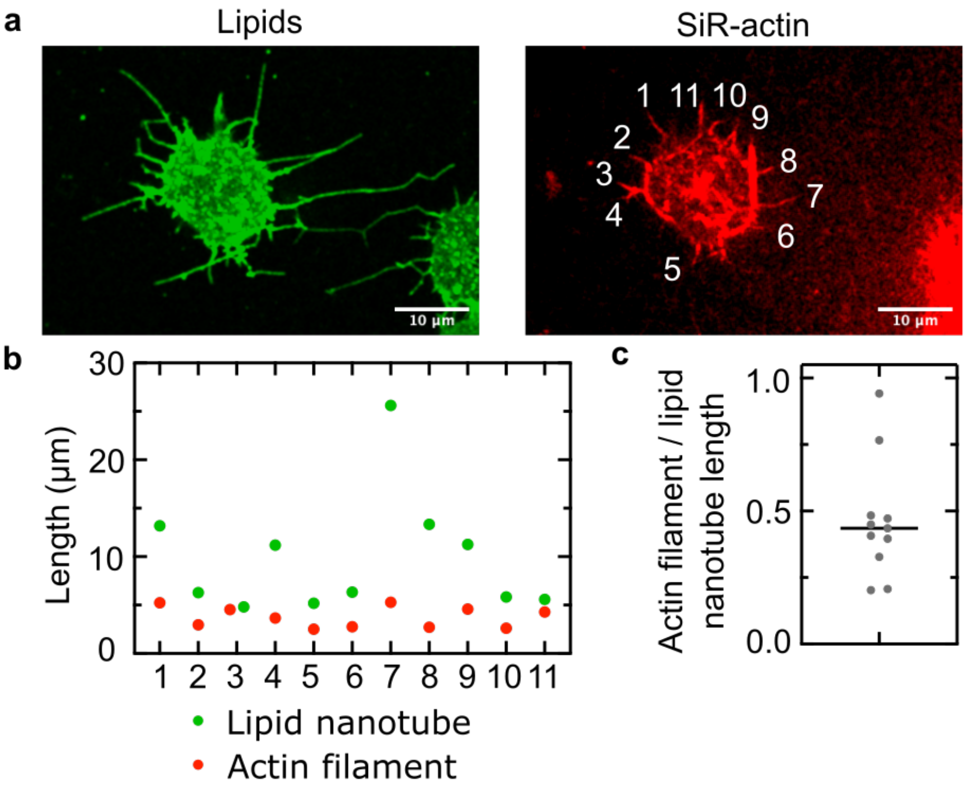

Figure S11: Characterization of actin filaments extending into lipid nanotubes. **a** Confocal image of a Jurkat cell (membrane labeled with WGA-Alexa488, $\lambda_{ex} = 488\,\text{nm}$) and cellular actin (red, labeled with SiR-actin $\lambda_{ex} = 640\,\text{nm}$) after acto-myosin assisted pulling of lipid nanotubes. Scale bar: 10 µm. **b** Length of eleven lipid nanotubes (green) and actin filaments (red) within the lipid nanotube. The corresponding nanotubes are marked in **a**. **c** Fraction of actin filament over lipid nanotube length (the black line indicates the median, n=11).

Supporting Figure S12: Mitochondria and lysosomes do not enter lipid nanotubes of Jurkat cells

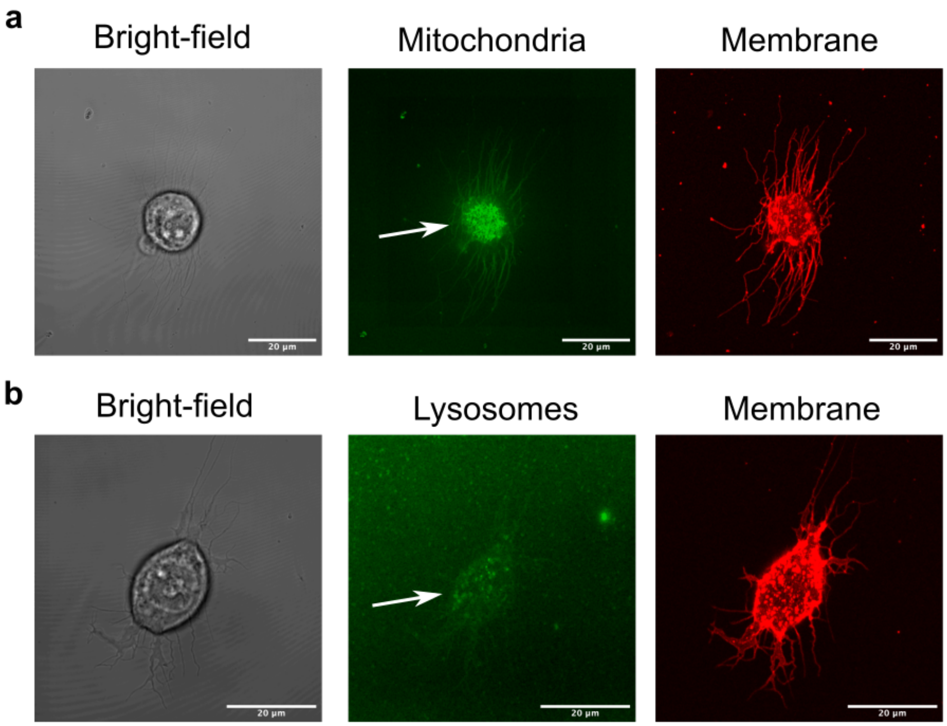

Figure S12: Mitochondria and lysosomes do not enter lipid nanotubes of Jurkat cells. **a**, **b** Confocal images of Jurkat cells (membrane labeled with WGA-Alexa647, $\lambda_{ex} = 647\,\mathrm{nm}$) stained for mitochondria (**a**, labeled via LysoTracker green DND-26, $\lambda_{ex} = 488\,\mathrm{nm}$) and lysosomes (**b**, labeled via MitoTracker FM green, $\lambda_{ex} = 488\,\mathrm{nm}$). Scale bar: 10 μm.

Supporting Figure S13: HaCaT cells do not form lipid nanotubes

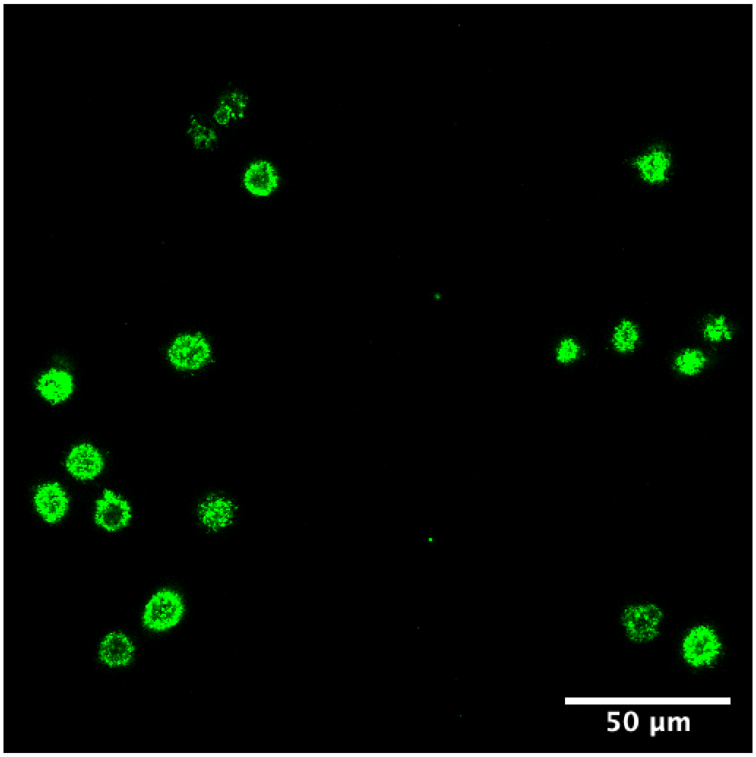

Figure S13: HaCaT cells do not form lipid nanotubes. Confocal image of HaCaTs in presence of 25 µM biotinylated cholesterol and aligned actin filaments on an HMM-coated substrate. Under these conditions we did not observe the formation of lipid nanotubes for Jurkat cells. Scale bar: 50 µm.

3 Supporting Videos

Supporting Video S1: Time series of random actin filaments

Confocal time series of rhodamine-labeled actin filaments ($\lambda_{ex} = 561\,\text{nm}$) in an *in vitro* motility assay over the course of 40 s. Actin filaments move randomly without preferred orientation.

Supporting Video S2: Time series of aligned actin filaments

Confocal time series of rhodamine-labeled aligned actin filaments ($\lambda_{ex} = 561\,\text{nm}$) in an *in vitro* motility assay over the course of 40 s. Actin filaments move parallel to each other in one preferred direction.

Supporting Video S3: Time series of aligned actin filament patterns

Confocal time series of rhodamine-labeled aligned actin filament patterns ($\lambda_{ex} = 561\,\text{nm}$) in an *in vitro* motility assay over the course of 10 min. Occasionally, aligned actin filament patterns emerge that form and move around local vortices. Scale bar: 50 µm.

Supporting Video S4: Time series of lipid nanotube dynamics after pulling from GUVs

Confocal time series of GUVs (membrane labeled with Atto488-DOPE, $\lambda_{ex} = 488\,\text{nm}$) functionalized with biotinylated lipids depicting the dynamics of lipid nanotubes over time at the substrate interface. Scale bar: 50 µm.

Supporting Video S5: Time series of lipid nanotube pulling from Jurkat cells

Confocal time series of Jurkat cells (membrane labeled with WGA-Alexa488, $\lambda_{ex} = 488\,\text{nm}$) functionalized with biotinylated cholesterol depicting the pulling of lipid nanotubes over time at the substrate interface. Scale bar: 50 µm.

Supporting Video S6: Displacement over time of a Jurkat cell during lipid nanotube pulling

Confocal time series of a Jurkat cell (membrane labeled with WGA-Alexa488, $\lambda_{ex} = 488\,\text{nm}$) during lipid nanotube formation. There is almost no displacement of the cell (indicated as red line) over the time course of imaging. Scale bar: 10 µm.

Supporting Video S7: Displacement over time of a GUV during lipid nanotube pulling

Confocal time series of a GUV (membrane labeled with DOPE-Atto488, $\lambda_{ex} = 488\,\text{nm}$) during lipid nanotube formation. There is almost no displacement of the cell (indicated as red line) over the time course of imaging. Scale bar: 10 µm.

Supporting Video S8: 3D projection of Jurkat cells with random actin and biotinylated cholesterol

Confocal 3D projection of a Jurkat cell (membrane labeled with WGA-Alexa488, $\lambda_{ex} = 488\,\text{nm}$) functionalized with biotinylated cholesterol depicting the pulling of lipid nanotubes at the substrate interface with random actin filaments.

Supporting Video S9: 3D projection of Jurkat cells with aligned actin and biotinylated cholesterol

Confocal 3D projection of Jurkat cells (membrane labeled with WGA-Alexa488, λ_{ex} = 488 nm) functionalized with biotinylated cholesterol depicting the pulling of lipid nanotubes at the substrate interface with aligned actin filaments.

Supporting Video S10: 3D projection of Jurkat cells with random actin and no biotinylated cholesterol

Confocal 3D projection of a Jurkat cell (membrane labeled with WGA-Alexa488, λ_{ex} = 488 nm) functionalized without biotinylated cholesterol depicting no pulling of lipid nanotubes at the substrate interface with random actin filaments.

Supporting Video S11: 3D projection of Jurkat cells with aligned actin and no biotinylated cholesterol

Confocal 3D projection of a Jurkat cell (membrane labeled with WGA-Alexa488, λ_{ex} = 488 nm) functionalized without biotinylated cholesterol depicting no pulling of lipid nanotubes at the substrate interface with aligned actin filaments.

Supporting Video S12: Actin filament dynamics during lipid nanotube pulling of a Jurkat cell (DOPE-Atto488)

Confocal time series of a Jurkat cell (membrane labeled with WGA-Alexa488, λ_{ex} = 488 nm) during lipid nanotube formation. Scale bar: 10 µm.

Supporting Video S13: Actin filament dynamics during lipid nanotube pulling of a Jurkat cell (SiR-actin)

Confocal time series of a Jurkat cell (membrane labeled with WGA-Alexa488, $\lambda_{ex} = 488\,\text{nm}$) during lipid nanotube formation with labeled SiR-actin (corresponding to Video S12). There does not seem to be any time delay in between the initial lipid nanotube pulling and actin filament presence within the lipid nanotube. Scale bar: 10 µm.

References

(1) Pardee, J. D.; Aspudich, J. *Methods in Enzymology*; Elsevier, 1982; pp 164–181.

(2) Kron, S. J.; Spudich, J. A. Fluorescent actin filaments move on myosin fixed to a glass surface. *Proceedings of the National Academy of Sciences* **1986**, *83*, 6272–6276.

(3) Margossian, S. S.; Lowey, S. *Methods in Enzymology*; Elsevier, 1982; pp 55–71.

(4) Rahman, M. A.; Salhotra, A.; Månsson, A. Comparative analysis of widely used methods to remove nonfunctional myosin heads for the in vitro motility assay. *Journal of Muscle Research and Cell Motility* **2018**, *39*, 175–187.

(5) Das, T.; Safferling, K.; Rausch, S.; Grabe, N.; Boehm, H.; Spatz, J. P. A molecular mechanotransduction pathway regulates collective migration of epithelial cells. *Nature Cell Biology* **2015**, *17*, 276–287.

(6) Ollech, D.; Pflästerer, T.; Shellard, A.; Zambarda, C.; Spatz, J. P.; Marcq, P.; Mayor, R.; Wombacher, R.; Cavalcanti-Adam, E. A. An optochemical tool for light-induced dissociation of adherens junctions to control mechanical coupling between cells. *Nature communications* **2020**, *11*, 472–472.

(7) Arganda-Carreras, I.; Kaynig, V.; Rueden, C.; Eliceiri, K. W.; Schindelin, J.; Car-

dona, A.; Sebastian Seung, H. Trainable Weka Segmentation: a machine learning tool for microscopy pixel classification. *Bioinformatics* **2017**, *33*, 2424–2426.

(8) Jaqaman, K.; Mettlen, M.; Kuwata, H.; Loerke, D.; Danuser, G.; Schmid, S. L.; Grinstein, S. Robust single-particle tracking in live-cell time-lapse sequences. *Nature methods* **2008**, *5*, 695–702.

(9) Angelova, M. I.; Dimitrov, D. S. Liposome electroformation. *Faraday Discussions of the Chemical Society* **1986**, *81*, 303.

(10) Schaller, V.; Weber, C.; Semmrich, C.; Frey, E.; Bausch, A. R. Polar patterns of driven filaments. *Nature* **2010**, *467*, 73–77.

(11) Schroer, C. F. E.; Baldauf, L.; van Buren, L.; Wassenaar, T. A.; Melo, M. N.; Koenderink, G. H.; Marrink, S. J. Charge-dependent interactions of monomeric and filamentous actin with lipid bilayers. *Proceedings of the National Academy of Sciences* **2020**, *117*, 5861–5872.

(12) Tinevez, J.-Y.; Perry, N.; Schindelin, J.; Hoopes, G. M.; Reynolds, G. D.; Laplantine, E.; Bednarek, S. Y.; Shorte, S. L.; Eliceiri, K. W. TrackMate: An open and extensible platform for single-particle tracking. *Methods* **2017**, *115*, 80–90.
